# Application of Flow Cytometry in Primary Immunodeficiencies: Experience From India

**DOI:** 10.3389/fimmu.2019.01248

**Published:** 2019-06-11

**Authors:** Manisha Rajan Madkaikar, Snehal Shabrish, Manasi Kulkarni, Jahnavi Aluri, Aparna Dalvi, Madhura Kelkar, Maya Gupta

**Affiliations:** Department of Paediatric Immunology and Leukocyte Biology, National Institute of Iummunohematology (ICMR), Mumbai, India

**Keywords:** flow cytometry, primary immunodeficiency disorders, familial HLH, severe combined immunodeficiency (SCID), chronic granulomatous disease (CGD)

## Abstract

Primary immunodeficiency diseases (PID) are a clinically and immunologically heterogeneous group of disorders of immune system. Diagnosis of these disorders is often challenging and requires identification of underlying genetic defects, complemented by a comprehensive evaluation of immune system. Flow cytometry, with its advances in the last few decades, has emerged as an indispensable tool for enumeration as well as characterization of immune cells. Flow cytometric evaluation of the immune system not only provides clues to underlying genetic defects in certain PIDs and helps in functional validation of novel genetic defects, but is also useful in monitoring immune responses following specific therapies. India has witnessed significant progress in the field of flow cytometry as well as PID over last one decade. Currently, there are seven Federation of Primary Immunodeficiency Diseases (FPID) recognized centers across India, including two Indian Council of Medical research (ICMR) funded centers of excellence for diagnosis, and management of PIDs. These centers offer comprehensive care for PIDs including flow cytometry based evaluation. The key question which always remains is how one selects from the wide array of flow cytometry based tests available, and whether all these tests should be performed before or after the identification of genetic defects. This becomes crucial, especially when resources are limited and patients have to pay for the investigations. In this review, we will share some of our experiences based on evaluation of a large cohort of hemophagocytic lymphohistiocytosis, severe combined immunodeficiency, and chronic granulomatous disease, and the lessons learned for optimum use of this powerful technology for diagnosis of these disorders.

## Introduction

Primary immunodeficiency diseases (PID) are an heterogeneous inherited group of disorders of different components within the immune system, resulting from genetic defects (1). PIDs are clinically and immunologically diverse and require a wide array of diagnostic tools for their accurate diagnosis. Flow cytometry, with the advances that have occurred in the last few decades, has emerged as an indispensable tool for evaluation of the immune system (2). It helps in enumeration as well as characterization of immune cells, and thus helps in the diagnosis of large number of PIDs.

It has now become widely available and is increasingly used for diagnostic purposes. In India, we have nearly 2000 installations of different flow cytometers across the country. The majority of them are being utilized for the measurement of CD4 counts in HIV infected patients and leukemia immunophenotyping. However, in the last decade there has been significant progress in the field of PID in India; currently there are two “Indian Council of Medical research-Centre of Excellence (COE) for PID” and seven Federation of Primary Immunodeficiency (FPID) centers across India. Both the COEs not only focus their work on setting up diagnostic facilities for PIDs in India, but also on understanding the immunopathogenesis of certain rare PIDs.

Due to the complexity of the immune system, multiple assays, which are expensive, are often required for comprehensive evaluation of the immune system. Moreover, they may not always give a definite diagnosis in spite of extensive evaluation. Furthermore, for final confirmation of diagnosis one always requires identification of the underlying genetic defect. With recent progress in the genetics field and increasing accessibility to Next Generation sequencing (NGS) based analysis, NGS is often used as a preferred modality for the diagnosis of PIDs. However, one needs to remember the utility and limitations of both genetic testing as well as phenotypic analysis. It is important that for all the genetic defects identified, corresponding immunological consequences are demonstrated, which are most often based on flow cytometry.

## Utility of Flow Cytometry for Diagnosis of PIDs- Experience at ICMR-NIIH

ICMR-NIIH is one of the few centers in India providing comprehensive workup for patients suspected with PID. To determine the utility of flow cytometry in the diagnosis of PIDs, we retrospectively correlated findings of flow cytometry based assays and molecular confirmation of the disorder. In the last 10 years, we have diagnosed 753 PID patients by using flow cytometry based assays including immunophenotyping, specific protein detection, and functional analysis. Of these patients, we could molecularly characterize 319 PID patients; 232 (73%) by using direct Sanger sequencing and 87 (27%) by using NGS.

In our experience, perforin deficiency (FHL-2) LAD-I and CGD were among the PIDs in which flow cytometry based assays provided a direct clue for the underlying genetic defect. Sanger sequencing of the respective genes correlated in >90% of the patients (LAD-I: 100%; FHL2 (Perforin deficiency): 97%; CGD: 90%). Thus, for molecular confirmation of these disorders, one should first perform Sanger sequencing of the respective genes rather than directly proceeding for NGS. This approach will be less time consuming and cost-effective.

However, for some PIDs, especially HLH with defective degranulation mechanism and SCID, multiple genes are involved. Flow cytometry based degranulation assay helps in identifying HLH patients with defective degranulation assay, and lymphocyte subset assay, T cell proliferation, and naïve T cell markers help in identifying SCID patients. Though flow cytometry based assays help in the diagnosis of these disorders, they are not sufficient for identifying specific genes involved in causing the disorder. Thus, these assays may be utilized as screening tests for diagnosing the disorders and molecular confirmation targeted at NGS is essential (Supplementary Table 1).

At ICMR-NIIH, we have maximum experience of using flow cytometry based assays in three disorders included under PIDs: familial hemophagocytic lymphohistiocytosis (FHL); severe combined immunodeficiency diseases (SCID); and chronic granulomatous diseases (CGD). We have reviewed the use of flow cytometry in these diseases systematically and shared some of our important practical experiences for the further, improved utility of flow cytometry in diagnosis of these disorders.

## Familial Hemophagocytic Lymphohistiocytosis (FHL)

Hemophagocytic lymphohistiocytosis (HLH) is a life-threatening hyperinflammatory syndrome, characterized by excessive activation of macrophages and T cells, resulting from defective cytotoxicity. It presents as a severe systemic illness with persistent high-grade fever, progressive cytopenias, and hepatosplenomegaly.

HLH can either be genetic (due to inherited defects in NK cell function) or acquired HLH (HLH resulting from secondary causes like infections, malignancy, etc.). Genetic HLH can be classified as familial HLH (FHL) and lymphoproliferative syndromes and FHL is then further sub-classified as with and without hypopigmentation. Till now, based on the mutated gene, FHL is classified as FHL2 (*PRF1*), FHL3 (*UNC13D*), FHL4 (*STX11*), and FHL5 (*STXBP2*) encoding for Perforin, Munc13-4, Syntaxin11, and Syntaxin binding protein 2, respectively (3).

FHL1 (9q21.3-22) was identified by homozygosity mapping of four inbred families of Pakistani origin (4), however, the disease-causing gene has so far not been identified in this locus. The incidence of the four types varies significantly in different ethnic groups. FHL with hypopigmentation includes Griscelli syndrome type 2 (GS2) (*Rab27a*), Chediak-Higashi syndrome (CHS) (*LYST*), and Hermansky-Pudlak syndrome type 2 (*AP3B1*). Exposure to EBV or other viruses can trigger HLH associated with lymphoproliferative syndromes, which can either be X-linked (XLP and XMEN) or autosomal recessive (*ITK* deficiency and CD27 deficiency).

Clinical and laboratory features of HLH overlap with those of severe infection and other inflammatory diseases, leading to either misdiagnosis or delay in the diagnosis of HLH. Unless specific genetic defect is identified, no single laboratory test is specific for HLH. Thus, in 1991, the Histiocyte society established guidelines for HLH diagnosis which collectively included clinical manifestations and laboratory findings. These criteria were revised in 2007 (5). Five of the eight criteria are required for diagnosis of HLH. Though these criteria help in the diagnosis of HLH, it does not help in differentiating genetic HLH from acquired HLH.

Both genetic and acquired HLH patients have impaired NK cells and CTL cells function. However, genetic HLH patients have inherited defect in the granule mediated cytotoxicity, while acquired HLH patients have transient defect. Thus, evaluating NK cell and CTL cell function in HLH patients helps in identifying genetic HLH patients.

Reduced NK cell cytotoxicity is one of the eight HLH diagnostic criteria (5) used for identifying HLH patients. New flow cytometry based assays have been developed in recent years, for evaluating NK cell and CTL cell functions by detection of intracellular perforin levels and degranulation assays (determined by upregulation of CD107a expression). Granule release assay (GRA) is a screening test for diagnosing FHL3, FHL4, and FHL5 patients, and helps in discriminate primary HLH with degranulation defects from secondary HLH. Whereas, detection of intracellular perforin levels helps in identifying FHL2 patients. Flow cytometry based assays, for determination of intracellular SAP and XIAP expression, helps in the diagnosis of XLP-1 and XLP-2, respectively.

In 2017, Rubin et al. retrospectively studied the diagnostic accuracy of NK cell cytotoxicity, intracellular perforin expression, and CD107a upregulation in a large cohort of HLH patients. In this, the sensitivity and specificity of these assays were evaluated in HLH patients. The authors concluded, firstly, the protocols used for NK cell cytotoxicity assay are labor intensive, usually involving radioactivity, and are not widely available. Secondly, the assay does not discriminate between primary and secondary HLH and, therefore, is not useful for differential diagnosis of HLH subtypes. It also proposes that perforin and CD107a tests are more sensitive and no less specific, compared with NK-cell cytotoxicity testing when screening for genetic HLH, and should be considered as an addition to current HLH criteria (6).

## Perforin and Granzyme Expression

Apoptosis of target cells recognized by NK cells and CTLs is induced by the synergistic action of perforin and granzymes. Perforin deficiency caused by mutation in the *PRF1* gene causes FHL2 and accounts for 20 to 50% of all FHL cases (5, 7). Whereas, elevated granzyme B in NK cells and CTLs is reported as a signature of immune activation in HLH patients, regardless of underlying genetic defect (8).

Flow cytometric detection of perforin in NK cells and CTLs has been reported in literature and is found to be a rapid and sensitive approach for the detection of perforin deficiency (9). Two antibody clones, δG9 and B-D48, are available for the detection of perforin. Clone δG9 detects the form of perforin found in the acidic milieu of the granules and clone B-D48 recognizes both the late form of perforin as well as its newly synthesized form. For clinical flow cytometric screening of perforin in HLH patients, clone δG9 is used unanimously (6, 9–11), whereas, clone B-D48 is used in *in-vitro* assays, especially in cytokine-staining (ICS) assays (12).

In healthy individuals, perforin expression in NK cells is reported to be more than 80%, irrespective of age; however, that increases with age in CTLs (9, 13). Our data is also in agreement of these findings. In perforin deficient patients, both NK cells and CTLs have either absent or partial perforin expression and need further confirmation by molecular testing of *PRF1* gene. The most detrimental *PRF1* gene mutations are usually associated with minimal or no protein expression, while compound heterozygous *PRF1* gene missense mutations may encode partially active perforin and are predominantly detected in older patients with milder clinical manifestations probably due to the residual perforin protein (7, 14, 15). In a study by Kogawa et al. (9), perforin expression in parents (heterozygous carriers) of perforin deficient patients was evaluated. It was observed that they had normal perforin expression in NK cells but with reduced mean fluorescent intensity. CTLs, though, had reduced perforin expression (9). However, the reason for this reduced perforin expression in heterozygous carriers of *PRF1* mutations remains unclear.

**Figure 1:**
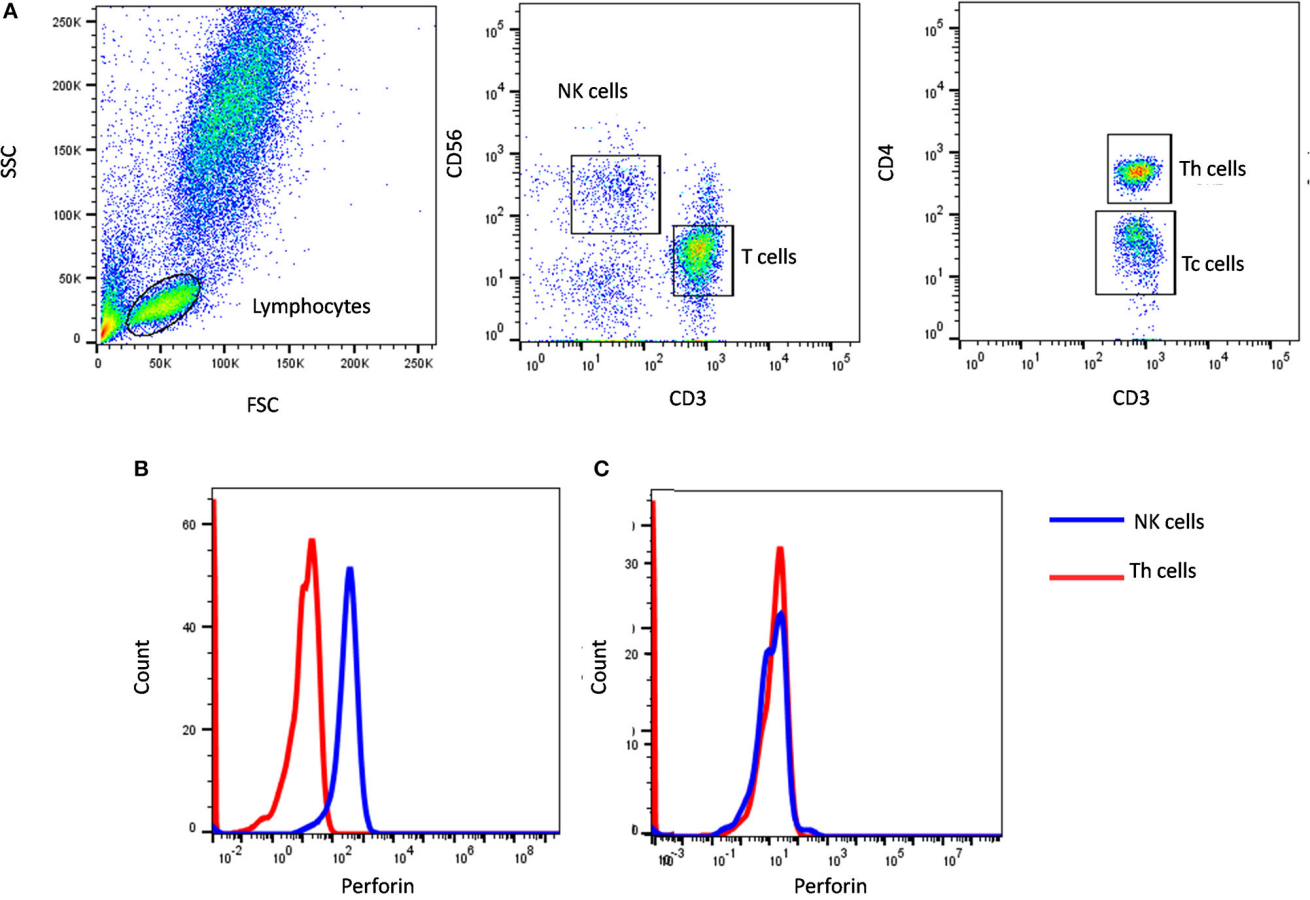
Perforin expression on NK cells. **(A)** Samples were analyzed by flow cytometry, gating on lymphocytes by forward/side scatter. Perforin expression was analyzed on natural killer (NK) cells (CD56+CD3-) (blue histogram) using T helper cells as internal negative control (red histogram). Perforin expression results are shown from **(B)** a healthy control and **(C)** FHL2 (Perforin deficient) patient.

In our institute, we have evaluated more than 600 HLH patients of which we have identified 39 perforin deficient patients. We could perform molecular characterization on *PRF1* gene in 36 patients and identified mutation in 34 patients (95%) (10) (Figure 1). Also, one HLH patient with normal perforin expression (78% on NK cells) harbored a mutation in the *PRF1* gene; thus highlighting that normal perforin expression does not rule out defect in functionally or structurally abnormal protein. In a recent report by Abdalgani et al. (11), where accuracy of perforin expression by flow cytometry was evaluated, it was concluded that, compared to patients with biallelic*PRF1* mutations, patients with monoallelic mutations, variants of uncertain clinical significance, and a minority of HLH patients without *PRF1* mutations, had normal perforin expression but lower mean fluorescence intensity (MFI) (11). Thus, while analyzing perforin expression, both frequency and MFI should also be considered. Overall literature and our experience support the fact that clinical flow cytometric screening for perforin deficiency is sensitive and is associated with a low false-negative rate.

Elevated granzyme B expression in NK cells and CTLs is a signature of immune activation in HLH patients, irrespective of genetic background. While assessing perforin expression, intracellular granzyme B staining is routinely utilized as an internal control in some laboratories. In 2013, Sabine reported granzyme B as a useful biomarker of disease activity (8). However, further studies including patients with MAS, autoinflammatory disorder, autoimmune diseases, sepsis, etc., are essential for understanding the utility of granzyme B in the diagnosis of HLH.

## NK Cell and CTL Degranulation Assay

Flow cytometry based granule release assay (GRA) is a rapid assay for the evaluation of granule exocytosis pathway. CD107a, a lysosomal protein, is present on the surface of cytolytic granules in NK cells and CTLs and is not expressed on the cell surface. After stimulation of these cells the lytic granules fuse with the plasma membrane of cytotoxic lymphocytes and CD107a is expressed on the cell surface. Abnormal degranulation after stimulation suggests defect in the degranulation mechanism, which includes cytolytic granule migration, docking, priming, or fusion.

This assay is a screening test for diagnosing FHL3, FHL4, and FHL5 patients, and helps in discriminating primary HLH with degranulation defects from secondary HLH.

In literature, various protocols for evaluating degranulation mechanism in NK cells and CTLs have been reported. These protocols mainly differ in the stimuli used, the initial sample used purified cells or whole blood cells and the use of a cytolytic content secretion inhibitor, monensin. Here we have reviewed the different protocols reported in literature and also shared our experience.

Different stimuli having specific mechanisms for activating CTLs and NK cells have been reported, including K562 (NK cell specific MHC I devoid target cells), PMA/ionomycin (protein kinase C activator), PHA (mitogen receptor), and anti-CD3 plus anti-CD-28 (TCR/CD3 complex). Although PMA/Ionomycin, PHA, and anti-CD3 plus anti-CD28 can all activate lymphocytes, PMA/ionomycin is reported to be the best one for short-term stimulation (16, 17). PMA is a substitute for diacylglycerol (DAG), one of the adaptor proteins required for the activation of protein kinase C, and Ionomycin increases intracellular calcium levels. Therefore, the combination of PMA/Ionomycin facilitates the activation of protein kinase C and an influx of intracellular calcium which are the necessary signaling events for degranulation.

Conventionally these assays use either PBMC or pure cell populations, however, this requires additional steps of cell purification and more volume of blood. In 2009, Claus M reported comprehensive analysis of NK cell function in whole blood samples (18). In this report, the CD107a surface expression was evaluated by stimulating NK cells with K562 cells using whole blood sample and no significant difference in the results were observed.

**Figure 2:**
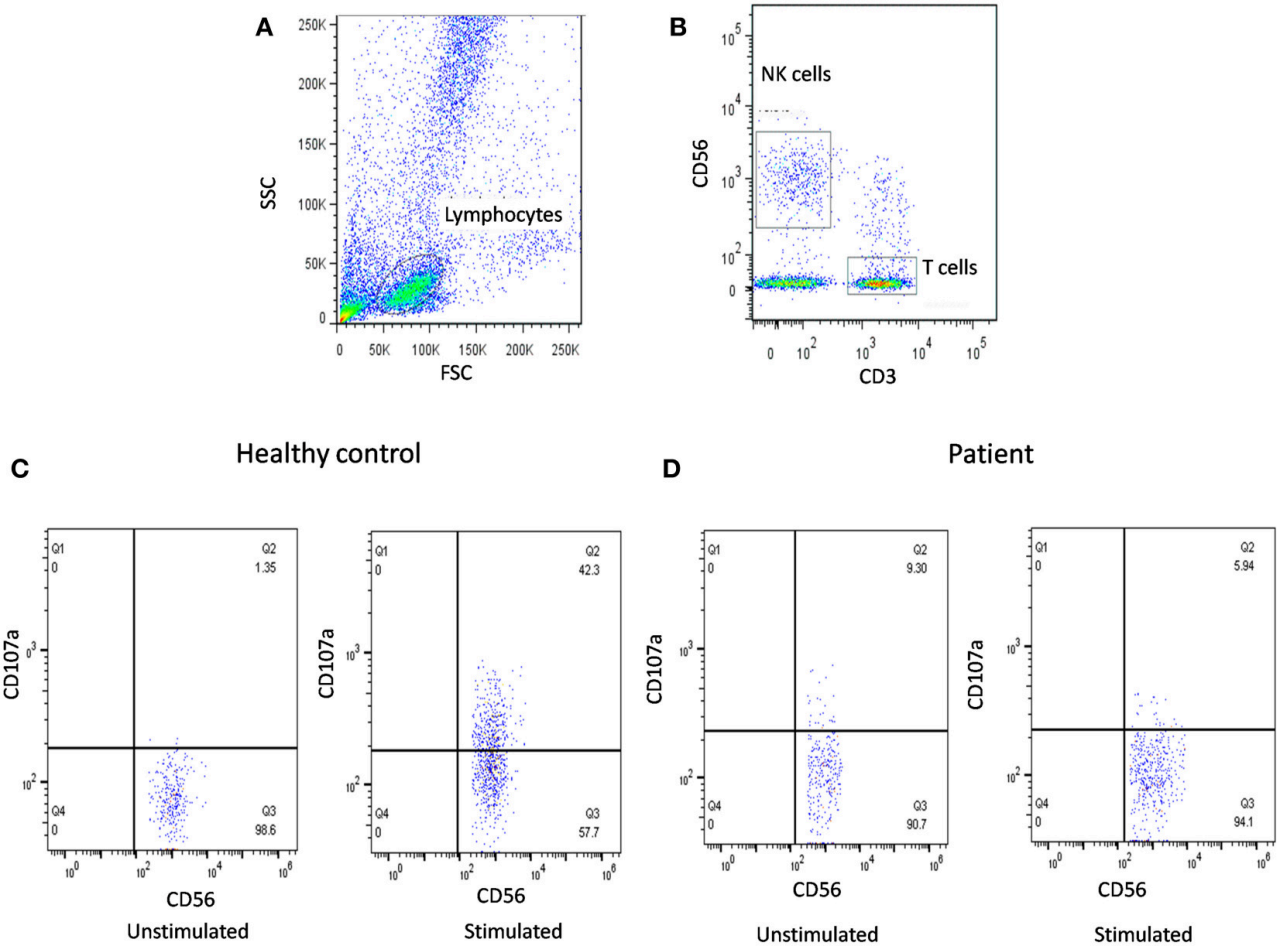
NK cell degranulation assay results. **(A)** Samples were analyzed by flow cytometry, gating on lymphocytes by forward/side scatter. **(B)** NK cells were gated based on (CD56+CD3-). CD107a expression was analyzed on unstimulated and Ca-I + PMA stimulated natural killer (NK) cells (CD56+CD3-). Degranulation assay results are shown from **(C)** healthy control (representative plot) and **(D)** FHL patients with degranulation defect.

In our previous publication (19), we have also reported NK cell degranulation assay using whole blood and stimulation with PMA/Ionomycin (Figure 2). We had compared PBMC and whole blood and also K562 and PMA/Ionomycin stimuli. The results of whole blood with PMA/Ionomycin stimuli showed comparable results to the conventional methods. In this modified assay, whole blood is used and no cell line is needed thus it requires reduced blood volume, is cost-effective, less time consuming, and avoids necessity for specialized laboratory for cell culture, which makes it applicable in routine clinical set-up.

After degranulation, once CD107a is expressed on the cell surface it becomes internalized by endocytosis. To prevent the degradation of internalized CD107a monensin is usually added. Monensin is a polyether ionophore that blocks the acidification of endocytic vesicles. However, such ionophores may disturb cellular signaling and function and should be avoided in experiments studying NK cell function. Moreover, in assays lasting 2 h or less, the internalization of surface expressed CD107a on resting NK cells is negligible (20, 21). Assays using monensin require 4–6 h of incubation, as reported by Bryceson YT (21); this was supported by our experience of CD107a expression as using a 2 h assay without monensin was observed to have comparable results to that reported in literature using monensin (19, 21).

Different reported studies have revealed that the results of degranulation on NK-cell and CTLs are in accordance with each other for the correct diagnosis of patients (22–24). However, in a recent report by Hori et al (25), it is suggested that, rather than NK-cell based assays, CD57+ CTL degranulation assay more effectively identified FHL-3 patients. Earlier studies have shown that CTL expressing CD57 has a high cytotoxic potential, and CD57 expression on CTL can be used as a measure of their degranulation capacity (25). This study concludes that NK cell degranulation assay detects FHL-3 patients with high sensitivity (100%) but low specificity (71%), whereas CD57+ CTL degranulation assay has high sensitivity and specificity (both 100%).

A low number of NK cells during active HLH episode often results in the interpretation of the assay result challenging. In such conditions, the CD107a degranulation on CTL for the diagnosis of HLH can be tested, as CTL numbers are usually much higher than NK cell numbers, even in patients with lymphopenia. Although limited studies are available in the literature on degranulation on CTLs as compared to NK cell mediated degranulation, a selective few reports suggest utilization of CTL degranulation for the characterization of FHL-3, FHL-4, and FHL-5 *in vitro* and *in vivo* (22, 25).

At our own institute, we have compared CD107a degranulation of NK-cells and CTL for the diagnosis of HLH in a small patient cohort (unpublished data). In our cohort, CD107a degranulation both on NK-cells and CTLs correlated in all patients. In nine patients we observed severe lymphopenia (absolute lymphocyte count <500/μl), all of whom had very low NK cell numbers (<25 cell/mm^3^); hence, performing NK cell degranulation was not feasible. These patients had normal CTL degranulation assays. Observations from our small cohort suggest that, in addition to decreased NK-cell degranulation being a diagnostic criterion for HLH, CTL degranulation assays can be used in those patients with a low NK cell number, although validation in large study cohort is needed for confirmation.

## SAP and XIAP Expression

For diagnosis of XLP-1 and XLP-2, all male patients should be additionally investigated for the expression of SAP (*SH2D1A*) and XIAP, respectively, (*BIRC4*).

XLP-1 and XLP-2 patients demonstrate low or absent SAP and XIAP expression, respectively (26–28). Since these are X-linked disorders, the bimodal pattern of SAP or XIAP expression in flow cytometric analysis can be used for the detection of carrier status, especially in mothers of affected patients (26). In carrier mothers, interestingly bimodal distribution of XIAP is seen to be skewed toward XIAP-expressing cells in all subsets, indicating a likely survival advantage for XIAP-expressing cells (28). *De novo* mutations in *SH2D1A* as well as *BIRC4* are also observed and in such cases bimodal pattern of respective protein might not be observed in carrier mothers.

However, molecular characterization of *SH2D1A* and *BIRC4* gene is essential for confirmation of diagnosis.

## Algorithm for Diagnosis of HLH

**Figure 3:**
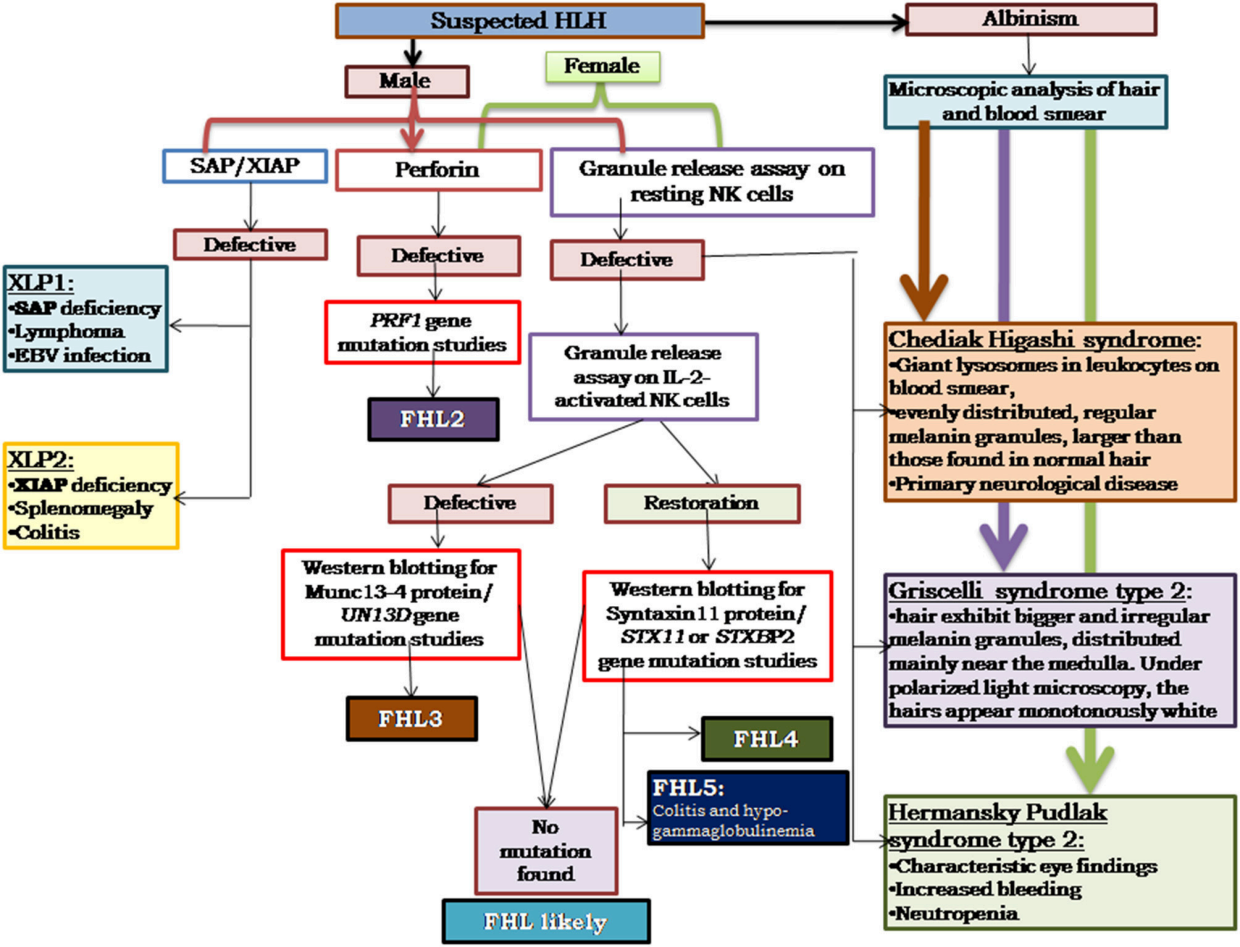
Illustrates the HLH algorithm based on the flow cytometric assays. All the patients fitting into HLH criteria, irrespective of age, and clinical presentations, should be screened for perforin expression and Granule release assay. All the male patients should be screened for SAP and XIAP expression. For patients clinically presenting with albinism, microscopic analysis of hair, and blood smear is essential for differential diagnosis of Chediak Higashi, Griscelli syndrome, and Hermansky-Pudlak syndrome. Based on the defect in expression of a particular protein identified, molecular characterization for the respective gene should be performed for confirmation of diagnosis (33).

Based on our experience, and also as reported in literature (29–33), perforin deficiency and granule release assay defect is observed to be more or less equally present and, hence, in our algorithm we suggest to perform perforin estimation and GRA simultaneously, which saves much time in diagnosis (Figure 3). It is also important to screen all suspected HLH patients using the flow cytometry based assays and all male patients for SAP and XIAP expression irrespective of age and clinical presentations.

## Severe Combined Immunodeficiency Diseases (SCID)

Severe combined immunodeficiency (SCID) is a highly complex and heterogeneous group of primary immunodeficiency disorders (PID). More than 30 different genetic defects can lead to SCID (34). A unifying feature of all the different forms of SCID is a defect in the T cell compartment. Depending on the genetic defect, the B cell, NK cell numbers, and/or function may also be affected.

**Figure 4:**
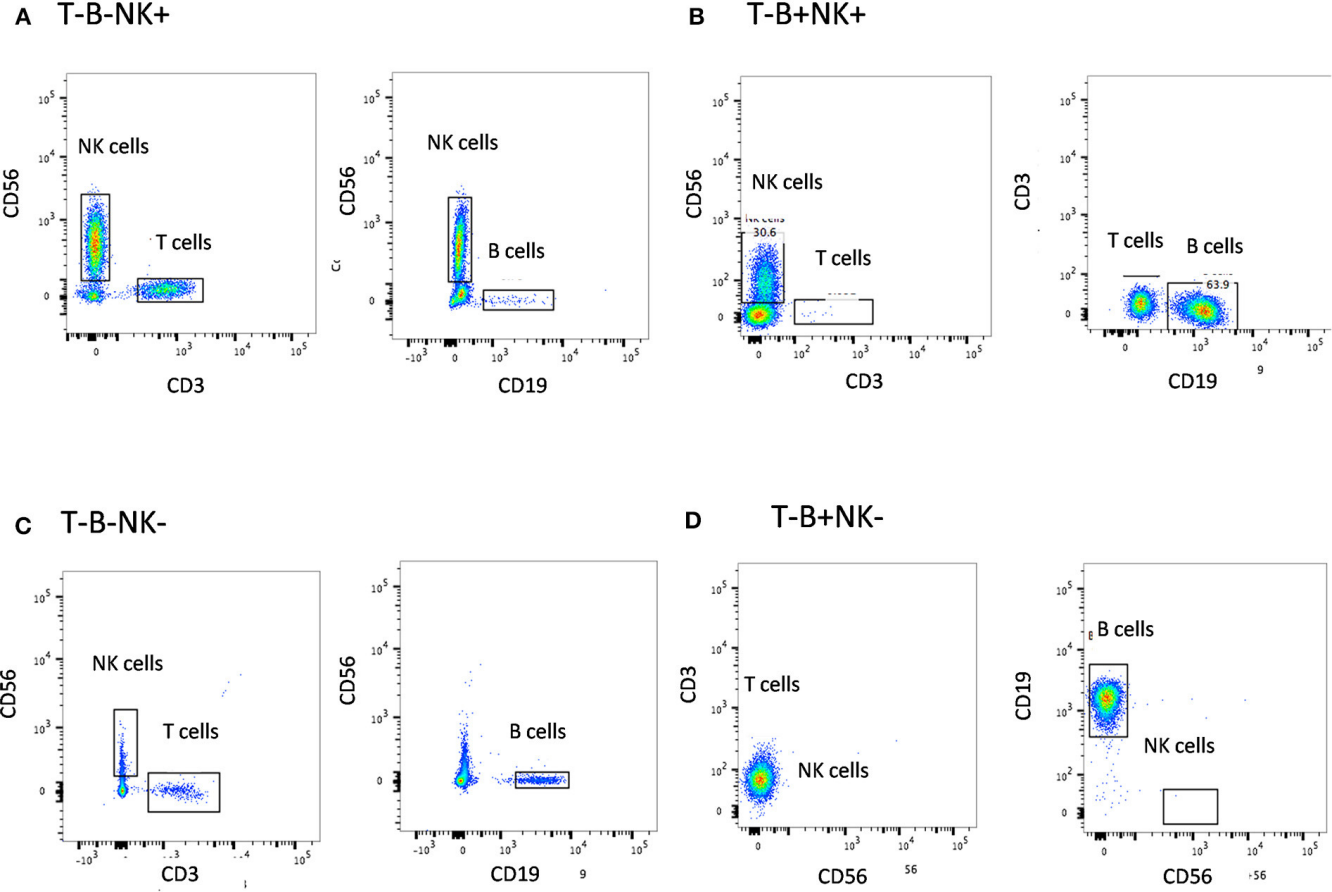
Lymphocyte subset analysis for SCID. Samples were analyzed by flow cytometry, gating on lymphocytes by forward/side scatter and gating T cells (CD3+), B cells (CD19+), and NK cells (CD56+CD3-) **(A)** T-B-NK+ **(B)** T-B-NK+ **(C)** T-B-NK- **(D)** T-B+NK- SCID, respectively.

**Figure 5:**
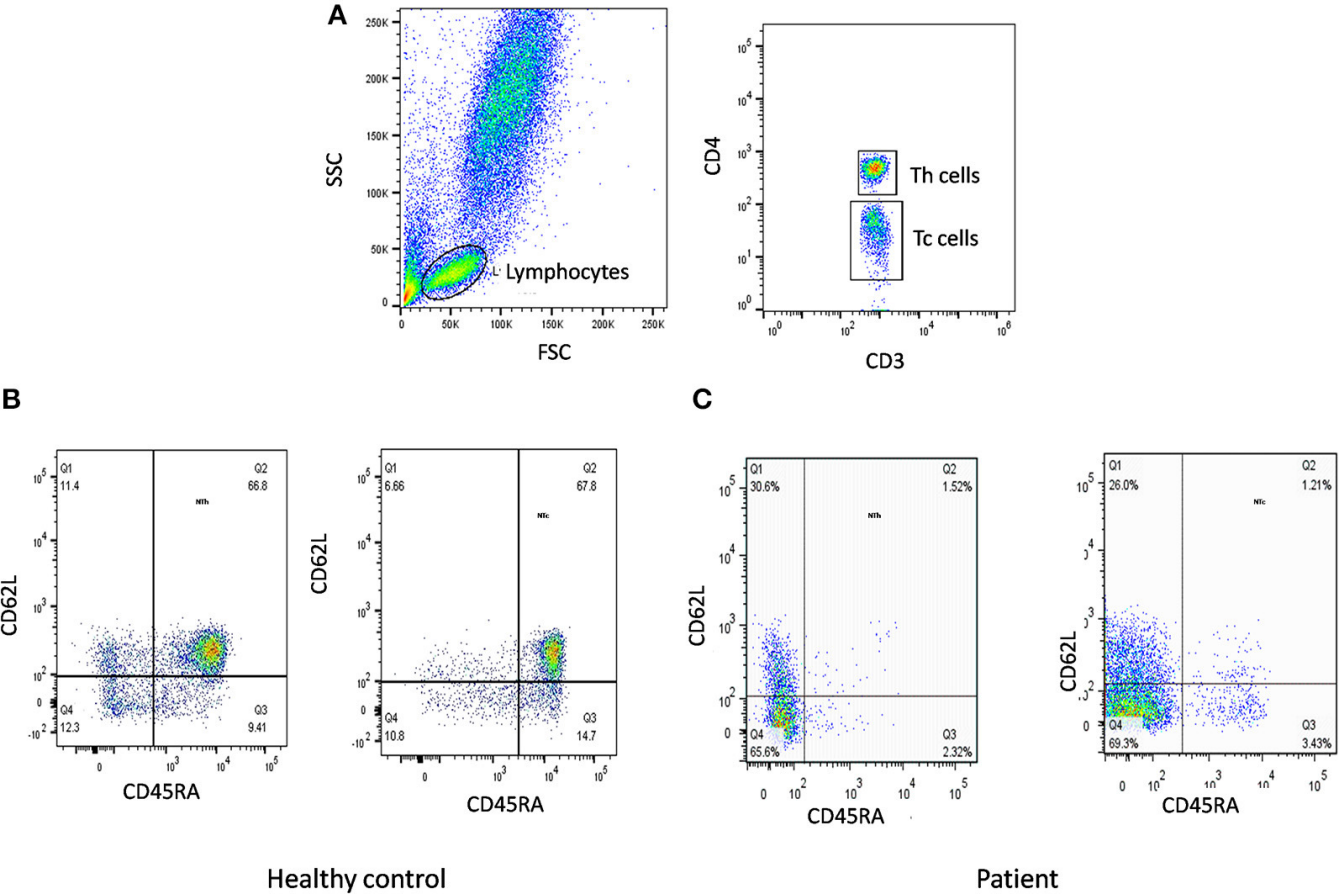
Naïve T cells analysis. **(A)** Samples were analyzed by flow cytometry, gating on lymphocytes by forward/side scatter. Tc and Th cells were gated as CD3+CD8+ and CD3+CD4+, respectively. Naïve T cells were gated as CD45RA+CD62L+ on Th cells and Tc cells. Figure represents Naïve T cell population in **(B)** healthy control **(C)** SCID patient. SCID patients had low percentages of naïve T cells.

The first step in diagnosis of SCID is the study of lymphocyte subsets (T, Th, Tc, B, NK) by flow cytometry for preliminary classification of SCID into T-B+NK+ SCID, T-B-NK+ SCID, T-B+NK- SCID, and T-B-NK- SCID (Figure 4). Typical SCIDs have absent/reduced (< 300 cells/μl) T cell counts (35). In cases with residual CD3+T cells (>300 cells/μL), the study of subsets that reflect their naïve/memory and activation state using CD45RA, CD45RO, and HLA-DR aids in the diagnosis of Leaky SCID and Omenn Syndrome. The different T cell transition states can be studied using a combination of CD45RA with CD62L or CD31 to study the naïve (C45RA^+^CD62L^+^), central memory (C45RA^−^CD62L^+^), and effector memory (C45RA^−^CD62L^−^) cells (Figure 5). The typical SCIDs lack the naïve T cell markers and both Leaky SCID and Omenn Syndrome patients have a predominance of CD45RO+ T cells. Varying display of the naive cell markers on CD4 and CD8 T cells may also give a clue to conditions with isolated T cell defects, such as selective deficiency of naïve Th cells in MHC class II deficiency. A highly activated state of the T cell hints at Omenn syndrome or maternal engraftment. Studying the lack of HLA-DR expression on T cells, B cells, and monocytes serve as a useful panel for the rapid identification of patients with MHC class II deficiency.

Genetic diagnosis of SCID is not straightforward because of an immunophenotypic overlap between different categories of SCID. A child with T-B+ SCID can have a defect in either *IL2RG, IL7RA*, or *JAK3* gene. Hence, in such cases, flow cytometric analysis of specific protein expressions may serve as a rapid tool to narrow down the list of possible genetic defect.

**Figure 6:**
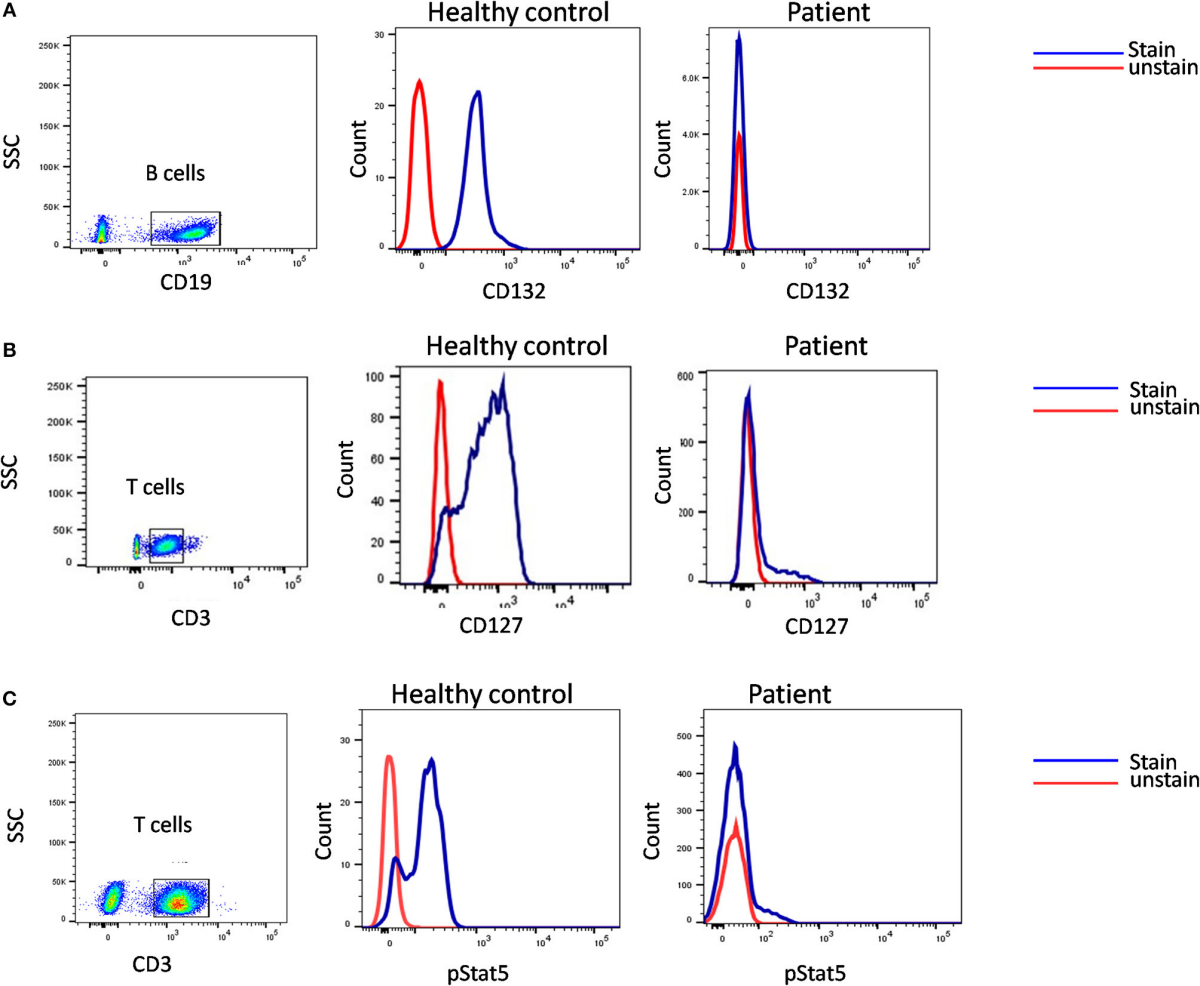
Samples were analyzed by flow cytometry, for **(A)** the common gamma chain (γc) (Or CD132), also known as interleukin-2 receptor subunit gamma or IL-2RG on CD19 cells. **(B)** IL7Ra (CD 127) expression on CD3 cells. CD132 and CD127 expression was absent in patient indicative of X –SCID and AR- IL7Rα. **(C)** Flow cytometric evaluation of STAT5 phosphorylation following IL-2 stimulation. Patient cells fail to respond. Healthy control (Blue histogram) and SCID patient (Red histogram).

A flow cytometry panel that defines subsets like CD132 (IL2RG), CD127 (IL7RA), CD3, and CD19 helps in classification of T-B+ SCID patients into X-Linked SCID or IL7RA deficient SCID (Figure 6). However, in cases with zero T cells, studying the T cell specific markers like CD127 holds a limited utility. Also, a reduced CD127 expression needs to be further evaluated for IL7 receptor internalization as high circulating levels of IL7 in lymphopenic patients can cause the down regulation of CD127. Flow cytometry also enables the study of intracellular proteins like phosphor-STATs. IL-2 stimulated JAK3-pSTAT5 expression on T cells helps identify JAK3 deficient SCID forms.

In our experience with the immunological characterization of SCID patients, almost 65% of our B^+^ SCID cohort had absent T cells. Hence, both CD127 and phospho-STAT5 assays had a limited utility in our study and we had to rely on genetic analysis to identify the defect (36). Exploiting the fact that CD132 is expressed on all the lymphocyte sub-populations, studying its expression on B cells helps rule out X-SCID. For patients that lack T cells, the use of IL-21 stimulant and analyzing the JAK3-pSTAT3 expression on B cells serves as an alternative method for the identification of JAK3 deficiency.

Generally, a T-B-NK- immunophenotypic pattern leads to a suspicion of Adenosine deaminase (ADA) deficiency. A flow cytometry based approach to assess the intracellular ADA levels can be performed to identify ADA deficient patients (37). The other possible defect is reticular dysgenesis (RD), which is suspected in the case of a defect in both the lymphoid and myeloid development. In our experience, we also identified a Purine nucleoside phosphorylase deficient (PNP) SCID child with an immunophenotypic pattern of T-B-NK–.

Within the T-B-NK+ SCID, it is extremely important to determine if the patient is sensitive to ionizing radiations. Recently, a flow cytometry based approach has been described which involves analysis of γH2AX as a useful marker for classifying patients with radiosensitive SCID (38).

Hypomorphic mutation in SCID genes leads to the generation of a residual number of T cells. In such cases, the basic flow studies have to be supported by T cell functional studies. These involve flow based assays, which use dyes like CFSE to monitor the T cell response to various stimulants like Phytohemagglutinin (PHA), anti-CD3, and anti-CD28 (39). Such assays are also useful in a setting of isolated T cells defects like ZAP70 deficiency, where patients lack CD8+ T cells but have normal CD4 cells which do not respond to CD3 stimulation.

The results of a T cell proliferation assay or tests that look for phosphor proteins is highly affected by the quality of the sample, especially when samples are shipped to a reference laboratory from different parts of the country. To demonstrate that the test results are not affected by the quality of the sample, it is mandatory to process the patient's sample along with a shipped healthy control sample. In cases where the result on healthy control sample seems inconclusive, a repeat testing needs to be performed on a fresh blood sample. In our experience, with samples that took 24–48 h to reach our laboratory it was difficult to establish a diagnosis in almost 20% of cases.

Another assay that holds importance in SCID patients with circulating T cells is the evaluation of T cell receptor diversity. This can be done by a PCR based approach called T-cell spectratyping, or a flow cytometry–based method that tests the Vβ repertoire. A restricted TCR repertoire is highly suggestive of SCID. In a few cases, testing for the TCRαβ+ T cells and TCRγδ+ T cells also helps identify specific defects in the development of T cells; for example, defects in the TCRa constant gene (TRAC), which show an impaired surface expression of TCRαβ complex (40). Similarly, flow based studies to look for specific CD3 subunits like CD3 zeta, help identify CD3 complex subunit deficiencies.

**Figure 7:**
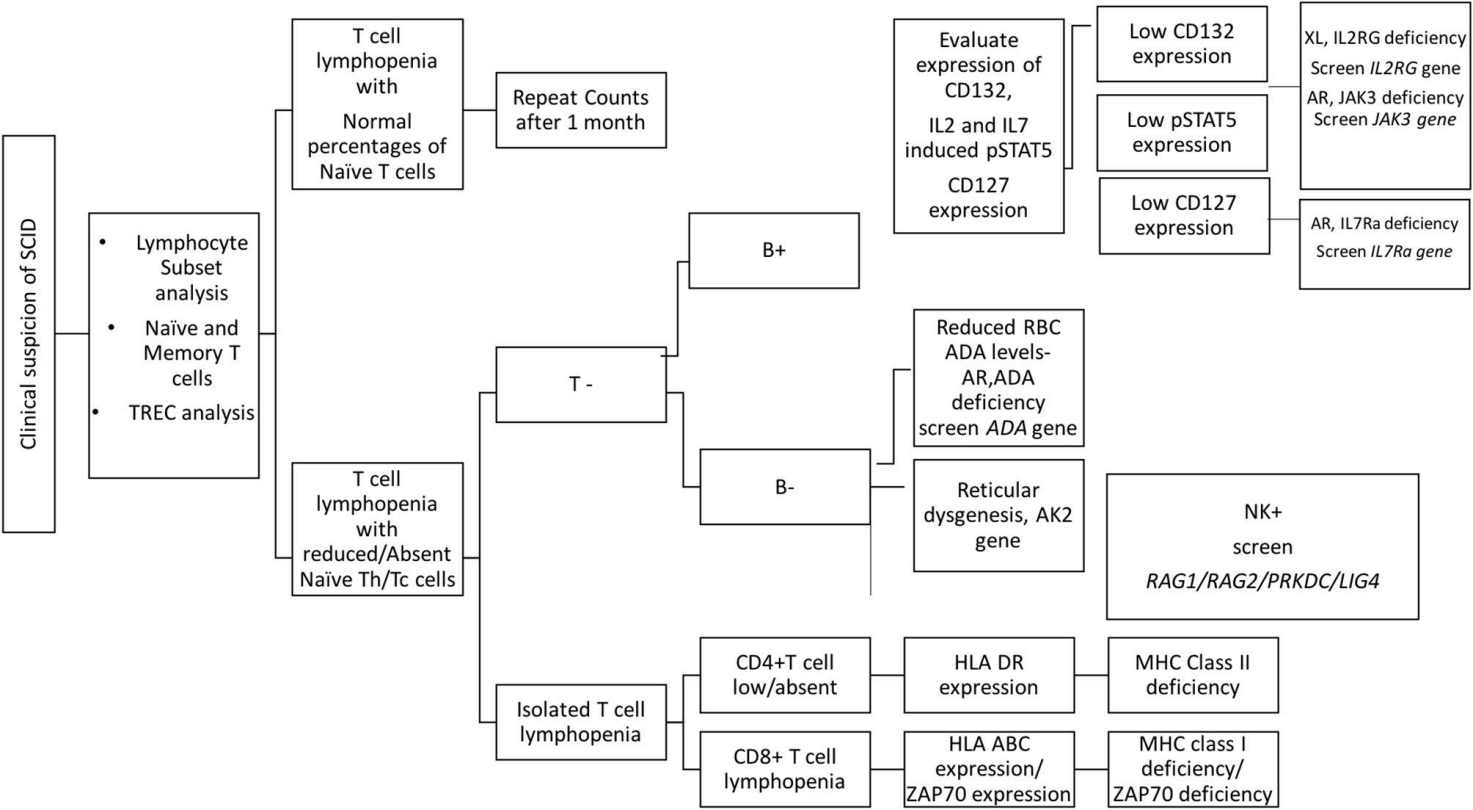
Diagnostic algorithm for diagnosis of SCID patients using flow cytometry based assays.

Overall, flow cytometry holds a great role in the immunological characterization of SCID by allowing enumeration of lymphocyte sub-populations, assessing thymic capabilities and the TCR diversity, measuring specific receptor expression, studying downstream molecules, and testing T cell function. It also serves as useful guide to determining the probable genetic defect involved in SCID pathogenesis (Figure 7).

## Chronic Granulomatous Diseases (CGD)

Chronic Granulomatous Disease (CGD), a rare (1: 200,000), inherited primary immunodeficiency disorder (PID), is caused due to a defect in components of NADPH oxidase complex (41, 42). Variability is observed in the pattern of underlying genetic defect with regards to the ethnicity and consanguinity. Overall, X-linked (XL)-CGD (due to mutations in *CYBB* gene) is the most prevalent (65%) type of CGD (42, 43). However, a higher rate of autosomal recessive (AR)-CGD (due to mutations in *CYBA, NCF1, NCF2, NCF4 genes*) is reported in the regions where consanguineous marriage is more common (44, 45). It is characterized by the inability of phagocytes to form reactive oxygen species (ROS) upon interaction with bacterial or fungal pathogens (46). The patients are susceptible to recurrent bacterial and fungal infections because of reduced or absent superoxide (47). The overlapping clinical manifestations and generic patterns in diagnostic tests sometimes mask the underlying genotype of CGD during an initial diagnosis. Significant differences are observed in age at diagnosis, residual superoxide activity, clinical course, and mean survival age among the CGD subtypes (41, 44, 48, 49).

Primary diagnosis of CGD involves laboratory screening tests that demonstrate an insufficiency of phagocytes to generate reactive oxygen species during respiratory burst activity. Nitroblue tetrazolium test (NBT) is a basic and most commonly used diagnostic test for CGD. However, it is now largely substituted with dihydrorhodamine assay (DHR) because of its sensitivity and specificity in providing rapid results (50). Both these tests are based on the principle of superoxide generation upon stimulation with agonists such as phorbolmyristate acetate (PMA) and N-Formyl-Met-Leu-Phe (FMLP), among others. DHR assay is performed by flow cytometry where formation of the reactive oxygen species (ROS) is monitored by fluorescence generated due to the oxidation of DHR 123 dye. It involves an indirect measurement of an ROS such as hydrogen peroxide (51, 52). The fluorescence generated after stimulation is quantitated by the mean peak channel fluorescence. The results are expressed as a stimulation index (SI) of neutrophils, which is a ratio of the mean fluorescence of stimulated neutrophils to the mean fluorescence of unstimulated neutrophils. Although, neutrophils are the cells of interest, when studying oxidative burst, monocytes serve as a low-level control and lymphocytes serve as a negative control. The SI value and coefficient of variation (CV) of the peak after the stimulation is extremely important to distinguish between the two genotypes, p47^phox^ deficiency and gp91^phox^ deficiency. The DHR assay is sensitive enough to detect trace amounts of residual superoxide activity which is retained in p47^phox^ deficiency. Hence, increased fluorescence (SI value or broad CV) is observed in p47^phox^ deficiency patients as compared to gp91^phox^ deficiency (50, 53). Thus, flow cytometry helps to identify the genotype-dependent variability in the evaluation of NADPH oxidase activity. It has been observed that autosomal recessive (AR-CGD) subtypes (*CYBA, NCF1, NCF2*, and *NCF4*gene defects) have slightly higher amounts of residual superoxide activity compared with the X-linked recessive (XL-CGD) subtype (*CYBB* gene defect) patients (48, 54). This mainly affects the severity of the clinical progression. However, previous studies have also shown that the amount of residual superoxide activity is dependent on the type of mutation in the affected gene (44, 48, 55). These diagnostic tests do not help to identify the exact genotype (except for the XL-CGD where mother is the carrier of the disease). In cases of AR-CGD and others where mothers do not show carrier pattern (~10%), additional tests are required to identify the genotype of CGD.

**Figure 8:**
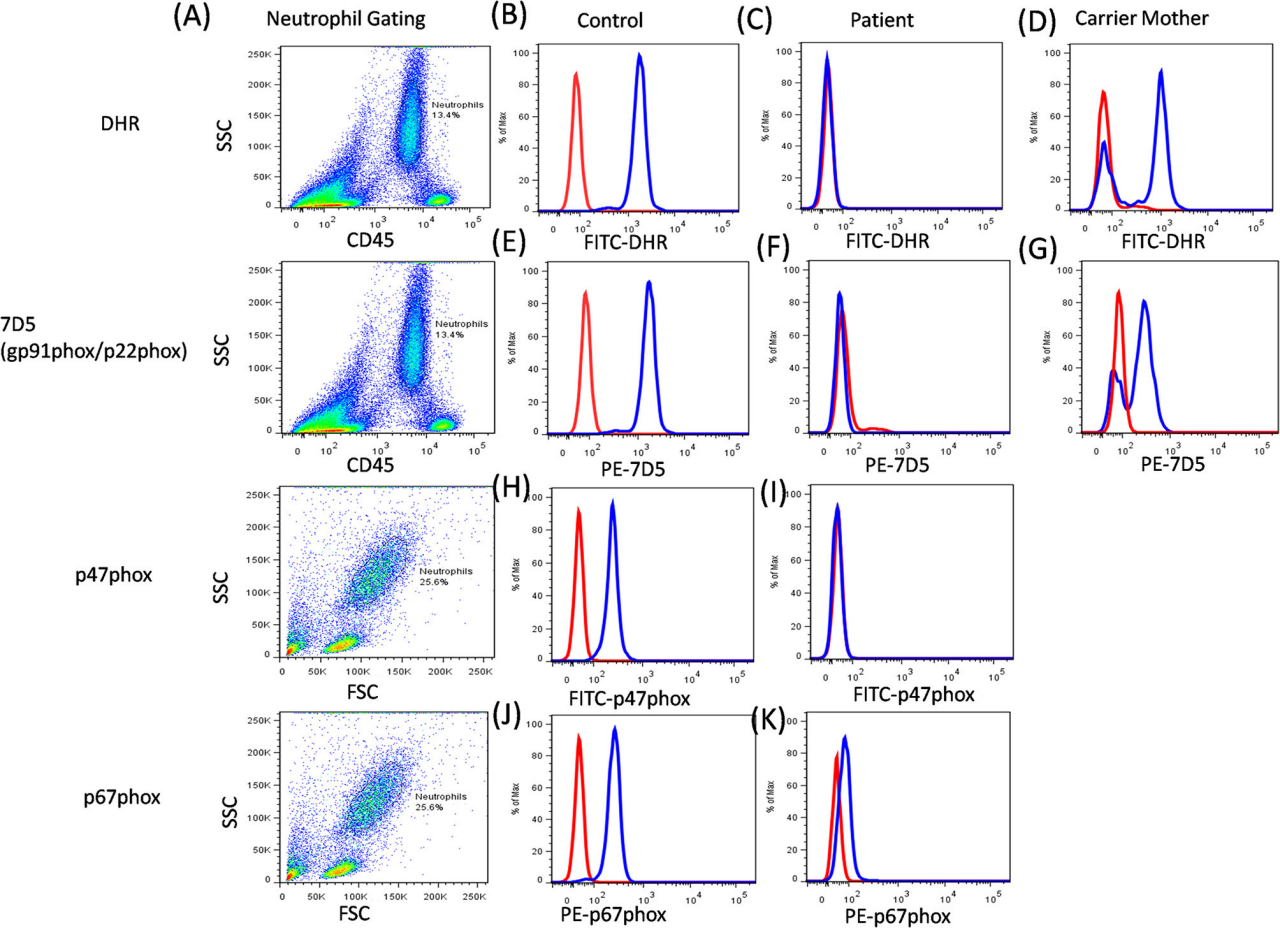
CGD analysis. **(A)** Samples were analyzed by flow cytometry, gating on neutrophils by forward/side scatter and/or CD45/side scatter. **(B)** a healthy control, **(C)** Abnormal DHR expression in CGD patient, **(D)** X-linked carrier mother-mosaic pattern. NADPH oxidase component expression (7D5 for anti-gp91phox/p22phox, anti-p47phox, and anti-p67phox) was analyzed on neutrophils cells. **(E)** a healthy control, **(F)** Abnormal expression of gp91phox (*CYBB* gene) or p22phox (*CYBA* gene) in CGD patient, **(G)** X-linked carrier mother-mosaic pattern. **(H)** a healthy control, **(I)** Abnormal expression of p47phox (*NCF1* gene) in CGD patient, **(J)** a healthy control, **(K)** Abnormal expression of p67phox (*NCF2* gene) in CGD patient.

Additionally, the functional analysis of NADPH oxidase complex involves component expression analysis by western blotting or flow cytometric evaluation, which involves the use of specific monoclonal antibodies (mAbs) against each of its components. Of the five components, gp91^phox^ and p22^phox^ form a heterodimer, which is expressed on the transmembrane. Other cytoplasmic components (p47^phox^, p67^phox^, and p40^phox^) form a heterotrimer and are translocated to the membrane upon activation (56). The study of specific protein expression by flow cytometry helps to identify defective protein components and also the underlying defective genotype (Figure 8).

The expression pattern of each of the component on neutrophils is studied, however, monocytes and B cells show low levels of expression, while T cells do not show any expression (57). The 7D5 mAb binds to an extracellular domain of gp91^phox^, as expressions of gp91^phox^ and p22^phox^ are dependent on each other for mature and stable expression. Hence, if any one of these proteins is defective then loss of expression of the other protein is also observed (58). Abnormal 7D5 expression can thus be inferred as a defect in the *CYBB* or *CYBA* gene, and requires further investigation or molecular confirmation. Similarly, an abnormal expression of other antibodies also suggests a defect in respective genes which further requires validation by sequencing the respective gene. One needs to keep in mind that the presence of protein expression does not necessarily implicate the presence/generation of a functional protein (45, 53). In this way, flow cytometry offers an opportunity to evaluate protein expression at a single cell level and is a secondary, helpful tool for further characterization of CGD patients.

Carrier detection is a very important aspect for genetic counseling and in prenatal diagnosis. Carriers of the XL-CGD could be detected by NBT and DHR analysis of mother's sample, which is not applicable in the case of AR-CGD (59). Additionally, the samples traveled from long distances or an old sample (more than 24 h old) make the analysis more difficult, due to non-viable neutrophils (60, 61). Studies by Kulkarni et al. (62) and Kuhns et al. (63) have described the use of flow cytometric NADPH oxidase component analysis for identification of the carriers of *NCF1* gene defect. Although molecular confirmation is the most preferred method to confirm the carrier status in both XL-CGD and AR-CGD, a quick identification of CGD patients and carriers is possible with NADPH oxidase component expression. The information on this is not conclusive due to limited data and there is a need for large patient cohort analyses.

Molecular confirmation is definitive diagnosis of CGD, however, it could be time-consuming and laborious to analyze each and every exon with the help of Sanger sequencing. The current technological advances, such as Next Generation Sequencing (NGS), could overcome these difficulties using targeted panel sequencing or Whole Exome Sequencing (WES) methods. Still, the factors such as long turn-around time and complication in data analysis due to presence of pseudogenes, big gene deletion, and/or GC rich regions are some of the challenges that makes it unsuitable as a routine diagnostic method (64, 65). Particularly, *NCF1* gene has two pseudogenes (>99% homology), which are difficult to analyze by either the Sanger or NGS method, and require a simple Gene Scan analysis to identify the most common Del GT mutation (at the beginning of exon 2) (66). In such cases, flow cytometric evaluation could play an important role in making the choice of appropriate method for further molecular characterization of CGD patients (44, 67).

**Figure 9:**
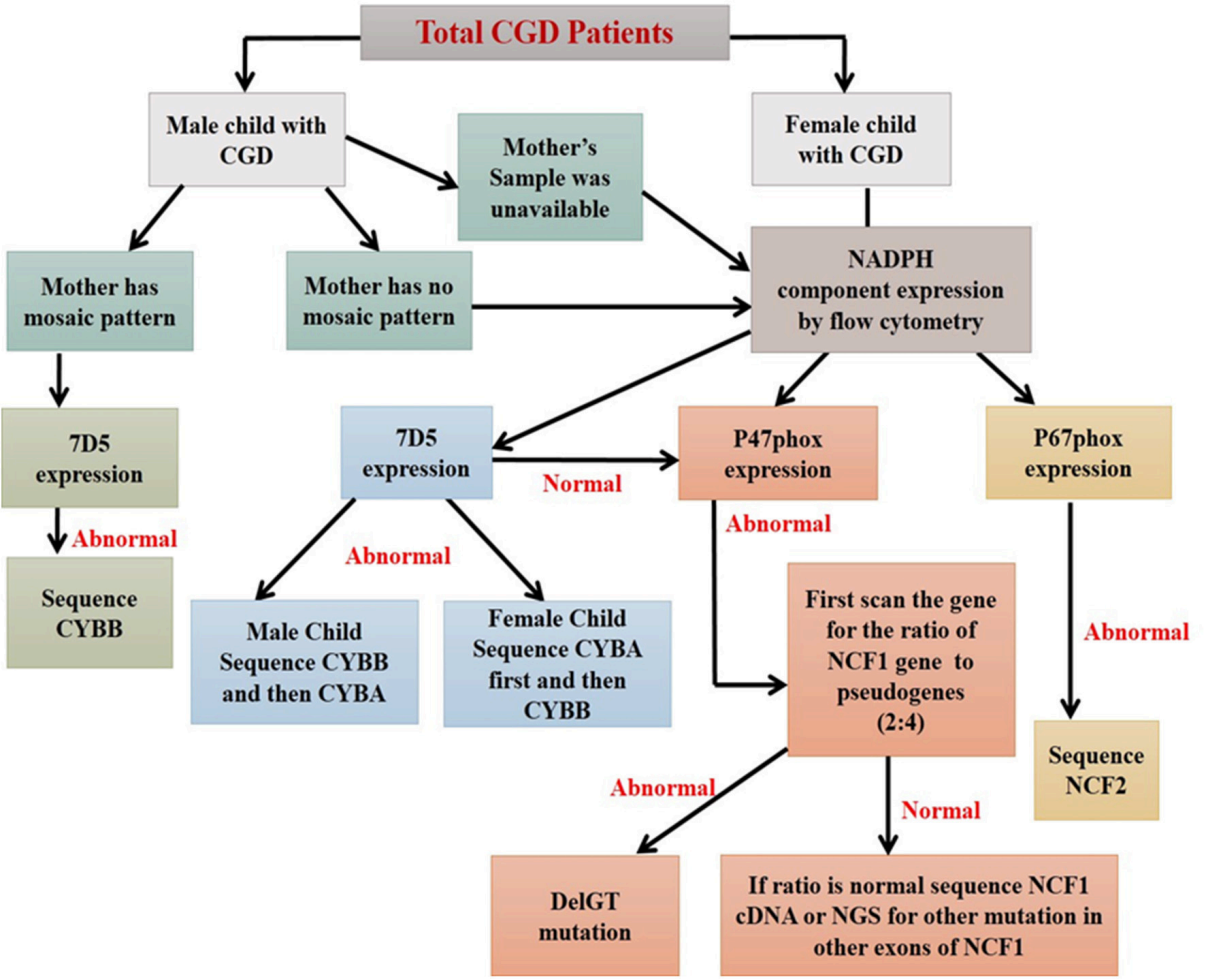
Algorithm for diagnosis of CGD patients using flow cytometry based assays (44).

This strategy was used in molecular characterization of 90 Indian patients, where only 12% (11 out of 90) of the patients required NGS analysis. Among them, a genotype could not be identified using flow cytometric evaluation in two patients. In a country like India with limited resources, the predominance of AR-CGD type, and a high frequency of consanguineous marriages, the flow cytometric classification of CGD patients remains a vital tool in the early diagnosis and guide for molecular characterization (by suggesting appropriate method) (44, 68) (Figure 9).

## Prenatal Diagnosis Using Flow Cytometry

Though, over the years, understanding of the pathogenesis of PIDs has improved, the management of these disorders still remains challenging. As these are inherited disorders, hematopoietic stem cell transplantation (HSCT) is essential for long term survival of majority of these patients. However, in country like India transplantation is often not feasible either due to lack of HLA-matched donor or the prohibitive cost of therapy. Thus, in such a scenario, genetic counseling with the possibility of carrier detection and prenatal diagnosis in affected families remains an important part of management.

Molecular diagnosis may not be possible or available in all affected cases; thus, phenotypic prenatal diagnosis by cordocentesis for families with an index case having an immunophenotypically well-characterized PID is an apt alternative. Thus, phenotypic prenatal diagnosis by flow cytometry offers a simple and rapid tool compared to molecular characterization (68, 69).

At ICMR-NIIH, we have established the normal ranges for the diagnostic parameters for a selected few PIDs, which include SCID, CGD, LAD-I, and XLA. In the last 10 years, using flow cytometry based assays, we offered prenatal diagnosis to 26 affected families (8 CGD, 9 SCID, 7 LAD-I, 1 XLA, and 1 MHC-II deficient) using a flow cytometry based assay of which five fetuses were affected with disease (unpublished data).

However, for utilizing flow cytometry as tool for prenatal diagnosis, there are few points one has to consider. Firstly, at the appropriate gestational age, the marker used for prenatal diagnosis should be expressed by a larger cell population. Secondly, gestational age-defined cut-off or range for the cell subset or marker of interest should be well-defined. Thirdly, the presence of a particular protein does not rule out functional defects and, hence, it can be used as a diagnostic marker in only those cases where the index case shows absent protein expression. And finally, one has to keep in mind that the use of flow cytometry based prenatal diagnosis has to be restricted to those PIDs in which diagnosis based on the markers and cells has been analyzed.

## Conclusion

Primary immunodeficiency disorders (PIDs) are an heterogeneous group of inherited disorders of the immune system. As the spectrum of PIDs is expanding, it is often difficult to diagnose PIDs based on clinical and conventional laboratory findings alone. Genetic analysis helps in the confirmation of diagnosis of a PID, however, they are expensive and time consuming. Flow cytometry, with its advances in the last few decades has emerged as an indispensable tool for enumeration and characterization of immune cells. Thus, this review has comprehensively evaluated our experience of the utility of flow cytometry in the diagnosis of PIDs, and concludes that flow cytometry serves as a bridge between clinical diagnosis and molecular testing, aiding rapid, and highly sensitive tools for the evaluation of PIDs. It not only provides clues to underlying genetic defects in certain PIDs, but also helps in the functional validation of novel genetic defects.

## Author Contributions

MM, SS, MKu, JA, AD, MKe, MG were involved in performing laboratory investigations and writing the manuscript. MM and SS compiled the data and reviewed the manuscript.

### Conflict of Interest Statement

The authors declare that the research was conducted in the absence of any commercial or financial relationships that could be construed as a potential conflict of interest.

## Supplementary Material

Supplementary Table 1 Total number of patients diagnosed phenotypically and genetically in 10 years at ICMR-NIIH.
