# Supplementary material for: Application of Flow Cytometry in Primary Immunodeficiencies: Experience From India
Source: Front Immunol. 2019 Jun 11;10:1248. doi: 10.3389/fimmu.2019.01248 (PMC6581000; doi:10.3389/fimmu.2019.01248)
Supplement: Supplementary Table 1 — Total number of patients diagnosed phenotypically and genetically in 10 years at ICMR-NIIH. [file Table_1.DOC]

| **Disease** | ***PHENOTYPIC DEFECT*** | **Inheritance** |  | **NO. OF PATIENTS** | | |
| --- | --- | --- | --- | --- | --- | --- |
|  |  |  | **Gene** | **SANGER** | **NGS** | **Total** |
| I. IMMUNODEFICIENCY AFFECTING CELLULAR AND HUMORAL IMMUNITY | | | | | | |
| X SCID | *T-B+NK-; CD132 deficiency* | X-linked | IL2RG | 6 | 3 | 9 |
| JAK3 deficiency | *T-B+NK- ;CD132+* | Autosomal Recessive | JAK3 | 0 | 6 | 6 |
| IL-7Ra deficiency | *T-B+NK+ ; IL7Ra Deficiency* | Autosomal Recessive | IL7Ra | 4 | 1 | 5 |
| RAG1/ RAG2 deficiency | *T-B-NK+ ; No Microcephaly* | Autosomal Recessive | RAG1/RAG2 | 6 | 3 | 9 |
| ZAP70 deficiency | *low CD8, CD4 low function* | Autosomal Recessive | ZAP70 | 1 | 1 | 2 |
| MHC class-II deficiency | *Absent MHC II expression* | Autosomal Recessive | *CIITA,* | 0 | 1 | 1 |
|  |  | Autosomal Recessive | *RFXAP,* | 0 | 3 | 3 |
|  |  | Autosomal Recessive | *RFX5,* | 0 | 1 | 1 |
|  |  | Autosomal Recessive | *RFXANK,* | 0 | 1 | 1 |
|  |  | Autosomal Recessive | *DCLRE1C* | 0 | 1 | 1 |
| ADA deficiency | *T-B-NK-, ADA Deficiency* | Autosomal Recessive | ADA | 3 | 1 | 4 |
| LRBA | *LRBA Deficiency, IgG and IgA Low* | Autosomal Recessive | LRBA | 0 | 3 | 3 |
| HIGM | *CD40 Deficiency, IgM Elevated* | Autosomal Recessive | CD40 | 1 | 0 | 1 |
|  | *CD154 Deficiency, IgM Elevated* | X-linked Recessive | CD40L | 10 | 0 | 10 |
| II. CID WITH ASSOCIATED OR SYNDROMIC FEATURES | | | | | | |
| PNP deficiency | *Low T cells* | Autosomal Recessive | PNP | 1 | 0 | 1 |
| WAS | *Thrombocytopenia, Increased IgA, IgE* | X-linked Recessive | WASP | 0 | 2 | 2 |
| HIGE | *IgE elevated, Reduced Switched and nonswtiched* | Autosomal Dominant | STAT3 (LOF) | 0 | 1 | 1 |
|  |  | Autosomal Recessive | EPG5 | 0 | 1 | 1 |
| III. PREDOMINANTELY ANTIBODY DEFICIENCY | | | | | | |
| XLA | *Absent* | X-linked Recessive | BTK | 15 | 1 | 16 |
|  | *or low B cel* | Autosomal Recessive | TCF3 | 0 | 2 | 2 |
| CVID | *Low IgG and IgM* |  | BAFF, TACI, ICOS | 0 | 0 | 0 |
| BENTA | *Bcell Lymphocytocysis* | Autosomal Dominant | CARD11 GOF | 4 | 1 | 5 |
| PIK3CD |  | Autosomal Dominant | PIK3CD | 0 | 1 | 1 |
| IV. DISEASE OF IMMUNE DYSREGULATION | | | | | | |
| Perforin deficiency | *Perforin deficiency* | Autosomal Recessive | PRF1 | 32 | 2 | 34 |
| Syntaxin11 deficiency | *Syntaxin11 deficiency* | Autosomal Recessive | STX11 | 2 | 2 | 4 |
| MUNC 13-4 deficiency | *MUNC13-4 deficiency* | Autosomal Recessive | UNC13D | 2 | 8 | 10 |
| STXBP2 deficiency | *STXBP2 deficiency* | Autosomal Recessive | STXBP2 | 0 | 2 | 2 |
| XIAP deficiency | *XIAP deficiency* | X-linked Recessive | XIAP | 0 | 4 | 4 |
| Griscelli syndrome 2 | *Cytopenia* | Autosomal Recessive | RAB27 | 0 | 2 | 2 |
| Chediak Higashi | *Neutropenia, cytopenia,* | Autosomal Recessive | LYST | 0 | 1 | 1 |
| ALPS |  |  | FAS | 0 | 0 | 0 |
|  |  |  | FASL | 1 | 0 | 1 |
| V. CONGENITAL DEFECTS OF PHAGOCYTE NO. FUNCTION OR BOTH | | | | | | |
| ELANE |  |  | ELANE | 0 | 2 | 2 |
| X-CGD | *Abnormal NBT DHR* | X-linked Recessive | CYBB | 26 | 1 | 27 |
| P47 defect | *Abnormal NBT DHR* | Autosomal Recessive | NCF1 | 40 | 2 | 42 |
| P67 defect | *Abnormal NBT DHR* | Autosomal Recessive | NCF2 | 2 | 3 | 5 |
| P22 defect | *Abnormal NBT DHR* | Autosomal Recessive | CYBA | 3 | 3 | 6 |
| LAD-I | *CD18/CD11a defect* | Autosomal Recessive | ITGB2 | 71 | 0 | 71 |
| GATA2 | *Low B, NK cells* | Autosomal Dominant | GATA2 | 0 | 1 | 1 |
| Severe Congenital Neutropenia |  | Autosomal Recessive | HAX1 | 0 | 1 | 1 |
| VI. DEFECT IN INTRENSIC AND INNATE IMMUNITY | | | | | | |
| MSMD | *CD212 Deficiency* | Autosomal Recessive | IL12RB1 | 0 | 7 | 7 |
|  | *CD119 Deficiency* | Autosomal Recessive | IFNgR1 | 2 | 2 | 4 |
|  | *Abnormal IFNgR2* | Autosomal Recessive | IFNgR2 | 0 | 3 | 3 |
|  | *low IL12p40 levels* | Autosomal Recessive | IL12p40 | 0 | 1 | 1 |
|  | *Abnormal pSTAT1 expression* | Autosomal Dominant | STAT1 | 0 | 2 | 2 |
| CMC | *Abnormal Dephosporylation* | Autosomal Recessive | STAT1 GOF | 0 | 2 | 2 |
| VII. AUTOINFLAMATORY | | | | | | |
| NOMID |  | Autosomal Dominant | NLRP3 | 0 | 1 | 1 |
| VIII. COMPLEMENT DEFICIENCY | | | | | | |
|  | *C1QA deficiency* | Autosomal Recessive | C1QA0 | 0 | 1 | 1 |
|  |  |  | **Total** | **232** | **87** | **319** |
